# Supplementary material for: Light-Responsive Soft Robot Integrating Actuation and Function Based on Laser Cutting
Source: Micromachines (Basel). 2024 Apr 16;15(4):534. doi: 10.3390/mi15040534 (PMC11051773; doi:10.3390/mi15040534)
Supplement: Supplementary file 1 [file micromachines-15-00534-s001.zip › Supporting Information.pdf]

Supporting Information

Light-Responsive Soft Robot Integrating Actuating and Function Based on Laser Cutting

Ben Jia, Changbo Liu, Yi Zhang, Yujin Tan, Xuecheng Tian, Yuanyuan Cui and Yuan Deng**

Note S1. Bending curvature calculation formula of the CNT-PDMS/PDMS/PI actuator

The parameters are defined as follows (shown in Figure S1):

L: The length of the CNT-PDMS/PDMS/PI actuator.

r: The radius of the arc of the curved CNT-PDMS/PDMS/PI actuator.

x: The horizontal displacement of the curved CNT-PDMS/PDMS/PI actuator.

y: The vertical displacement of the curved CNT-PDMS/PDMS/PI actuator.

θ/2: The chord tangent angle of the curved CNT-PDMS/PDMS/PI actuator.

θ: The bending angle of the arc of the curved CNT-PDMS/PDMS/PI actuator.

The curvature is defined as the reciprocal of radius (1/r).

The chord tangent angle is given by

$$\frac{\theta}{2} = tan^{-1}\frac{\mathrm{y}}{\mathrm{L}-\mathrm{x}} \qquad (1)$$

As the bending angle is given by

$$\theta = \frac{L}{r} \quad (2)$$

The curvature 1/r is deduced as

$$\rho = \frac{1}{r} = \frac{\theta}{L} \quad (3)$$

Hence, the curvature of the actuator can be calculated by using the vertical moving distance, horizontal moving distance, and self-length of the actuator under near-infrared illumination.

Note S2. Modeling the mismatched expansion structure.

TIMOSHENKO's beam theory[1] is always used to explain the photo-thermal expansion mechanism. According to the theory,

$$\rho = \frac{6E_1E_2h_1h_2(h_1+h_2)(\propto_1 - \propto_2)\Delta T}{(E_1h_1)^2 + (E_2h_2)^2 + 2E_1E_2h_1h_2(2h_1{}^2 + 3h_1h_2 + 2h_2{}^2)}$$

ρ represents the deformation curvature of the actuator; E_1 and E_2 are the elastic modulus of inert layer (PI layer) and active layers (CNT-PDMS layer and PDMS layer), respectively; h_1 and h_2 are the thicknesses of the inert layer and active layers, respectively; α_1 and α_2 are the CTEs of inert layer and active layers, respectively; and ΔT is the temperature change.

Note S3. Finite Element Simulation of Actuator Deformation.

Based on elasticity theory and stationarity principles, we have established a finite element analysis (FEA) simulation model. Finite element stimulations (Abaqus) are used to analyze the deformation process of bilayers with different scribing trenches patterns. In the simulation, an elastomer model with a size of 1:1 to the actual sample is imported by CAD drawing. The relevant parameters, such as thermal expansion coefficient, elastic modulus, and thermal conductivity, are measured by experiments

(Table S1). Each layer is connected by Tie constraints. According to the experimental measurement, the temperature rise of the material in the light process is about 100℃. Therefore, in order to simplify the model, we ignore the influence of thermal radiation and thermal convection, and choose the thermal conduction model. We use a fixed temperature field instead of the temperature generated by the laser spot irradiation sample (the temperature difference is measured by an infrared thermal imager). For grid division, we use hexahedral grids for single-layer structures without laser cutting, and we use tetrahedral grids for single-layer structures with laser cutting microgrooves. After the simulation calculation, the color of the nephogram shows the stress of the structure at this point.

Note S4. Finite element simulation of actuator heat conduction.

Based on the geometric size, the three-dimensional (3D) unit in COMSOL are utilized, the cube element is generated. In the simulation, a model with an actual sample size of 1: 1 is used. The groove width is 0.5 mm. The thermal conductivity is measured by experiments. A circular heat source with a fixed temperature is used instead of a laser spot (the spot temperature is measured by an infrared camera). The temperature boundary condition is defined. The external temperature is room temperature (293.15 K) and the heat transfer coefficient of the film and air is 25 W/m·k. The structure is divided into extremely refined grids and the results are studied by steady state. The color of the cloud shows the temperature of the structure at this point.

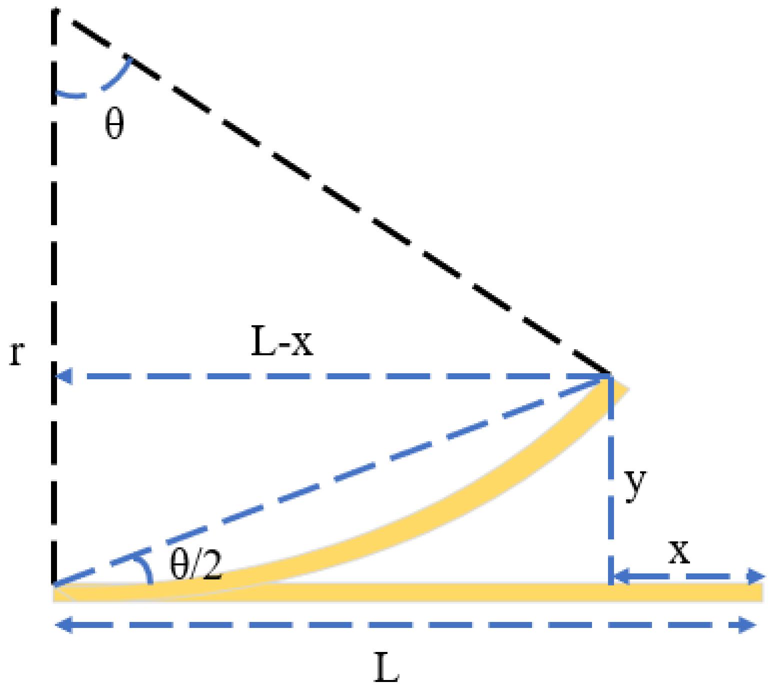

Figure S1. CNT-PDMS/PDMS/PI actuator with correlative parameters for calculating the bending curvature.

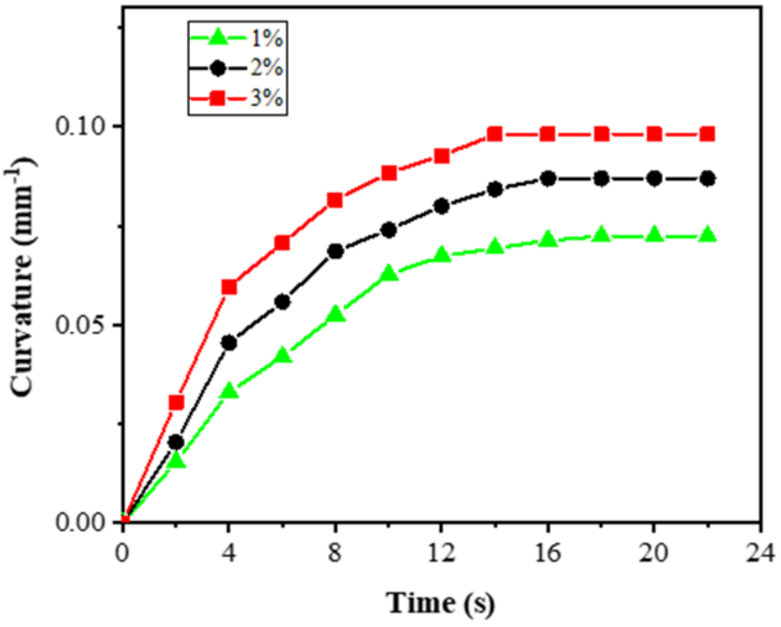

Figure S2. The time-curvature curve of three layers of film with different CNT content under light.

No.	Film	Thermal Expansion Coefficient (K^{-1})	Elastic Modulus (Mpa)	Heat Conductivity Coefficient (W/m·k)
1	CNT-PDMS	3.07E-4	1.87	0.227
2	PDMS	3.75E-4	1	0.16
3	PI	3.3E-5	1100	0.15

Table S1. Physical properties of each monolayer film.

Movie S1. Light-driven rolling of actuators with different groove and cutting line toward vertical NIR light illumination.

Movie S2. light-driven crawling of actuators without cutting in a process of switching vertical NIR light.

Movie S3. light-driven crawling of actuators with different cutting line depth in a process of switching vertical NIR light.

Movie S4. Crawling actuation of the actuator loaded with LEDs in a process of switching vertical light irradiation.

1. S. Timoshenko, Analysis of Bi-Metal Thermostats. *J. Opt. Soc. Am.* **11**, 233-255 (1925).
